# Patterns of Intimate Partner Violence: a study of female victims in Malawi

**DOI:** 10.5249/jivr.v5i1.139

**Published:** 2013-01

**Authors:** Shahrzad Bazargan-Hejazi, Sarah Medeiros, Reza Mohammadi, Johnny Lin, Koustuv Dalal

**Affiliations:** ^a^College of Medicine, Department of Psychiatry, Charles R. Drew University of Medicine and Science, Los Angeles, CA, USA.; ^*b*^David Geffen School of Medicine, Department. Psychiatry & Biobehavioral Sciences, Semel Institute, University of Cal-ifornia, Los Angeles, CA, USA.; ^*c*^College of Medicine, Charles R Drew University & David Geffen School of Medicine, University of California, Los Angeles, CA, USA.; ^*d*^Division of Social Medicine, Department of Public Health Sciences, Karolinska Institutet, Sweden.; ^*e*^Department of Psychology, University of Los Angeles, CA, USA.; ^*f*^Department of Public Health Sciences, University of Skovde, Sweden.

**Keywords:** Intimate Partner Violence, Physical Violence, Emotional Violence, Sexual Violence, IPV Exposure, Sub-Saharan Africa, Malawi

## Abstract

**Background:**

The term “intimate partner violence” (IPV) encompasses physical, sexual and psychological violence, or any combination of these acts, and globally is the most common type of violence against women. This study aims to examine the lifetime prevalence of different types of intimate partner violence (IPV) among Malawi women ages 15 to 49, and its association with age, education, and living in rural versus urban areas.

**Methods:**

Data was obtained from a cross-sectional study of data as part of the 2004 Malawi Demographic and Health Survey. Women were eligible for the study if they met the following criteria: 1) lived in one of the 15,041 households randomly selected from 522 rural and urban clusters located in 10 large districts of Malawi; 2) were married or cohabitating; and 3) were between the ages of 15 and 49 years. Consenting, eligible women responded to a comprehensive questionnaire covering demographic factors, health issues, as well as items related to physical, emotional and sexual IPV. To assess bivariate associations, chi-squared tests and multivariate logistic regressions were conducted.

**Results:**

Among the 8291 respondents, 13% reported emotional violence; 20% reported being pushed, shaken, slapped or punched; 3% reported experiencing severe violence, such as being strangled or burned, threatened with a knife, gun or with another weapon; and 13% reported sexual violence. Data showed women ages 15 to 19 were significantly less likely to report emotional IPV, women ages 25 to 29 were significantly more likely to report being pushed or shaken, slapped or punched (OR 1.35; CI: 1.05-1.73), and women ages 30 to 34 were significantly more likely to report sexual IPV, compared to women ages 45 to 49 (OR 1.40; CI: 1.03-1.90). Finally, women who had no ability to read were less likely to report sexual IPV than their counterparts who could read a full sentence (OR 0.76; CI: 0.66-0.87).

**Conclusions:**

The prevalence of different types of IPV in Malawi appears slightly lower than that reported for other countries in sub-Saharan Africa. Further studies are needed to assess the attitudes and behaviors of Malawi women towards acceptability and justification of IPV as well as their willingness to disclose it.

## Introduction

The term “intimate partner violence” (IPV) is used to encompass physical, sexual and psychological violence, or any combination of these acts.^1^ Globally, IPV is the most common type of violence against women^2^ and its worldwide prevalence is estimated to be between 10 and 75%.^3,4^ Existing reports also demonstrate that IPV is prevalent problem among men, though to a lesser degree,^5-7^ and is more frequent among men who have sex with men^8^ and those who abuse alcohol and drugs.^9,10^

Numerous risk factors have been shown to be associated with female victimization from IPV, including socio-demographic variables,^6,11,12^ length of stay in a relationship with a male partner, partners’ substance abuse, early intercourse, and childhood experiences of sexual abuse and/or IPV in the home.^13^ These risk factors demonstrate that IPV is multidimensional, and as conceptualized in a social-ecological framework, points to the important interplay of individual, family, community, and societal level factors. 

IPV is not only the product of the aforementioned risk factors, but also produces physical, mental, and social harm to its victims. It is associated with a broad range of physical and psychological consequences, including STDs,^14^ reproductive health issues,^1,15,16^ depression,^17^ PTSD,^18,19^ maternal death;^20^ difficulty with daily activities, memory loss, stress, suicidal thoughts/attempts, and even suicide.^21-24^

Recently, there has been an increasing focus on IPV in sub-Saharan Africa, likely related to a greater interest in human rights issues, as well as a greater understanding of the individual and societal consequences of violence. In Uganda, the prevalence of acts of IPV by a male partner against a female partner, during the year before the survey, was found to be 40% for verbal abuse and 30% for physical threats or violence.^13^ A study of Kenyan women revealed significant rates of exposure to emotional (24%), physical (38%) and sexual (14%) abuse, with many of these women experiencing reproductive consequences as a result of IPV.^16^ However, little research has been done to assess the lifetime prevalence and patterns of IPV in Malawi.^25^ It is important to understand the patterns of IPV and societal norms specific to the Malawi populace^26^ in order to develop effective interventions.^2^ Results of one study suggested that about 11% of Malawi women had experienced acts of IPV in the past year and 70% considered violence against women to be a serious problem in their community.^27^ In another study with a sample of 3,546 Malawi women, experiencing physical violence was reported to be the most common type of IPV in the household, and 30% of women reported physical IPV by their partner. In the same study, 25% of women reported experiencing emotional violence and 18% reported sexual violence.^28^

Malawi is a small, predominantly agricultural, landlocked country in east sub-Saharan Africa with a population of about 12 million and a life expectancy of just over 40 years.^29^ Attempts to modernize Malawi have continued since 1994, but for the most part, the country remains a traditional and patriarchal society attempting to develop within a global capitalist economy with limitations on its citizens in terms of educational and employment opportunities. In a country with a limited manufacturing base, Malawian women in particular are more likely to experience economic hardship and poverty. Consequently, many women in Malawi move to urban areas seeking jobs, usually domestic work, including cooking, cleaning, and childcare, which are difficult and poorly compensated.^30^ Economically disadvantaged women experience more stress and tension in close relationships than their less disadvantaged counterparts. This, in addition to working in urban areas, heightens the population’s risk for disproportional experience of IPV.^31-34^

There is also convincing evidence to suggest an association between lower educational attainments with an increased likelihood of experiencing IPV in women.^35,36^ Findings from a large population-based sample also support this claim.^37,38^

Socioeconomic factors are not the only factors contributing to the etiology of IPV, but failing to consider their significance can misinform interventions designed to alleviate domestic violence directed toward women.^34,39^

The current study aims to examine the lifetime prevalence of different types of intimate partner violence (IPV) in Malawi women ages 15 to 49, and its association with age, education, and living in rural vs. urban areas. Based on similar studies in the region, it is expected that the prevalence of IPV among women in Malawi will differ according to socio-demographic variables and geographic locations. Specifically, more acts of IPV will be reported by a) younger vs. older women; b) women with lower vs. higher levels of education; and c) women living in rural vs. urban areas. Results of this study may suggest directions for interventions aimed at reducing IPV among the vulnerable women in Malawi.

## Methods

The data was obtained from a cross-sectional study as part of the Malawi 2004 Demographic and Health Survey (MDHS). The MDHS was undertaken to provide information for policy makers, planners, and researchers for the development, monitoring and evaluation of health programs in Malawi. 

**Study Sites/Recruitment**

The participants in the MDHS were recruited from 15,041 households using two-stage systematic sampling, allowing for generalization of results to a larger Malawian population. These households were selected from 522 clusters (458 rural areas and 64 urban areas) in 10 large districts of Malawi: Mulanje, Thyolo, Kasungu, Salima, Machinga, Zomba, Mangochi, Mzimba, Blantyre, and Lilongwe. From the selected households, 12,229 women ages 15 to 49 were identified as eligible for the individual interview. The final sample consisted of 11,968 women who completed interviews for the entire survey (response rate, 96%).

A group of trained interviewers conducted face to face interviews in the respondent’s household between October 2004 and January 2005. Interviews were conducted in the native languages Chichewa and Tumbuka. Subsequently, trained staff at the National Statistical Office (NSO) entered the data. Questionnaires were translated and back-translated to avoid language error/barriers. Since collecting data on IPV is challenging, interviewers were instructed to read a script that would inform participants of the objective of the survey and the sensitive and personal nature of the IPV related questions. Interviewers were also instructed to proceed with the interview only when maximum privacy was ensured. If privacy was interrupted, they were instructed to either move to another section of the survey or stop the interview altogether. Securing privacy was ensured by conducting interviews within closed rooms and without the presence of any third person. Furthermore, the IPV items were placed towards the end of the survey to give interviewer an opportunity to build rapport with the interviewee. 

**Inclusion/Exclusion Criteria and Study Sample **

Women were eligible for the study if they were married or cohabitating (living/sharing the same household with a partner) during the time of interview, and were between the ages of 15 to 49 years. Those who did not meet the inclusion criteria were excluded from the study. Refusing to have sex with a man is on the World Health Organization (WHO) list of acts that may ‘trigger’ violence.^40^ Sexually active women between the ages of 15 and 49 are potentially subject to acts of IPV if they refuse to have sex with their partner, boyfriend, or husband. Although, they may be victims of IPV, including sexual IPV, regardless of this behavior. Based on these inclusion criteria, a total of 8,291 women were selected for the current study.

**MDHS Survey**

The MDHS survey includes data on the demographic and socioeconomic backgrounds of the women, as well as their reproductive history, knowledge and use of family planning methods, maternal health care, child care and nutrition, marriage and sexual activity, knowledge of HIV/AIDS, and report of IPV. For the current study, we used the data related to IPV.

**Outcome Variables**

Emotional, physical, and sexual acts of IPV were measured by the modified version of the Conflict Tactic Scale (CTS).^41^ This scale has been used in previous studies.^42,43^ The original 19-item scale was developed by Straus,^44^ and includes specific acts of violence such as slapping, punching, and kicking.^45^ The modified version^16^ consists of ten yes or no items. Emotional violence (2 items) was measured by asking respondents, since they turned, if they had ever been humiliated or threatened by their husband or partner. Physical violence was assessed by asking respondents if their husband/partner had ever pushed, shaken or thrown something at the respondent; slapped or twisted their arm; punched them with a fist or something harmful; and/or kicked or dragged them (4 items). Severe physical violence was assessed by asking respondents if their husbands or partner had ever tried to strangle or burn them; threatened them with a knife, gun or other weapons; or attacked them with a knife, gun or other weapons (3 items). Finally, sexual violence (1 item) was assessed by asking respondents whether or not their husbands/ partners had ever forced them into unwanted sexual intercourse or other sexual acts. In a multi-side IPV study conducted by the World Health Organization (WHO), the Cronbach's alpha, a measure of internal consistency or reliability across a set of items, collapsed across all 15 sites was calculated to be 0.81 (emotional violence), 0.66 (physical violence), and 0.73 (severe physical violence).^46^

**Predictor Variables**

We used the following demographic variables as the main predictors for the study: age, place of residence, education, and literacy level. Age brackets included ages 15 to 19, 20 to 24, 2, 5 to 29, 30 to 34, 35 to 39, 40 to 44, and 45 to 49. Place of residence was categorized as rural or urban. Education level included four categories: no education, primary school only, secondary school, and/or some form of higher education. Finally, respondents were grouped into one of the following categories for literacy level: ‘unable to read at all,’ ‘capable of reading a partial sentence,’ or ‘capable of reading a full sentence’. The survey received ethical approval from the Institutional Review Board of Opinion Research Corporation (ORC), Macro International Incorporated. Informed consent was obtained from the participants prior to interview, and the right to withdraw was emphasized throughout the survey. 

**Data Analysis**

We used chi-squared tests of association to assess relationships between socio-demographic factors and acts of IPV. A Bonferroni correction was used to reduce the possibility of Type I error from multiple comparisons in the chi squared test. We also used multivariate logistic regression analyses to assess the independent associations between socio-demographic variables and acts of IPV. The magnitude and direction of associations is expressed in odds ratios (ORs) with 95% confidence intervals (CIs). For all the statistical analyses, a significance level of p < .05 was employed. All data analysis was conducted using PASW (SPSS 18).

## Results

The overall characteristics of the respondents are displayed in Table 1. Fifty percent of the respondents were between 20 and 29 years of age. The smallest numbers of respondents were in the youngest and oldest age groups (9.1% and 6.5%, respectively). This is consistent with data showing that 67% of women ages 15 to 19 in Malawi are unmarried (have neither a husband nor cohabiting partner) and therefore ineligible for this study,^47^ and data indicating an average life expectancy of 41 years for both men and women in 2004.^48^ A majority of the women lived in rural areas (88%), which is consistent with the 2008 reports of a total urban percentage of 19% and an annual rate of change of 5%.^49^ Over half the women (62%) had a primary school education, while only 0.3% had higher education. This is comparable with studies showing that Malawians received an average of nine years of schooling in 2004.^49^ The percentage of women who could read a full sentence was 47%, which approximates data from 2003 showing a national literacy rate of 50% among women.^49^

**Table:** Table 1: **Overall Characteristics of the Sample (n = 8,291).**

Variable	Frequency (%)
Age (years)	
15-19	753 (9.1%)
20-24	2257 (27%)
25-29	1887 (23%)
30-34	1295 (16%)
35-39	874 (11%)
40-44	687 (8.3%)
45-49	538 (6.5%)
Place of Residence	
Urban	970 (12%)
Rural	7321 (88%)
Level of Education	
None	2316 (28%)
Primary	5176 (62%)
Secondary	772 (9.3%)
Higher	27 (0.3%)
Literacy Level	
Can’t Read	3680 (44%)
Partial Sentence	747 (9.0%)
Full Sentence	3850 (47%)
Type of IPV	
Emotional	12.5%
Being pushed or shaken, slapped or	19.9%
punched Severe Physical	2.8%
Sexual	13.3%

The prevalence of IPV include: emotional violence (13%), being pushed or shaken, slapped or punched (as one category) (20%), severe physical violence (3%) and sexual violence (13%).

**Bi-variate Associations**

**IPV and Age**

**Figure 1:**
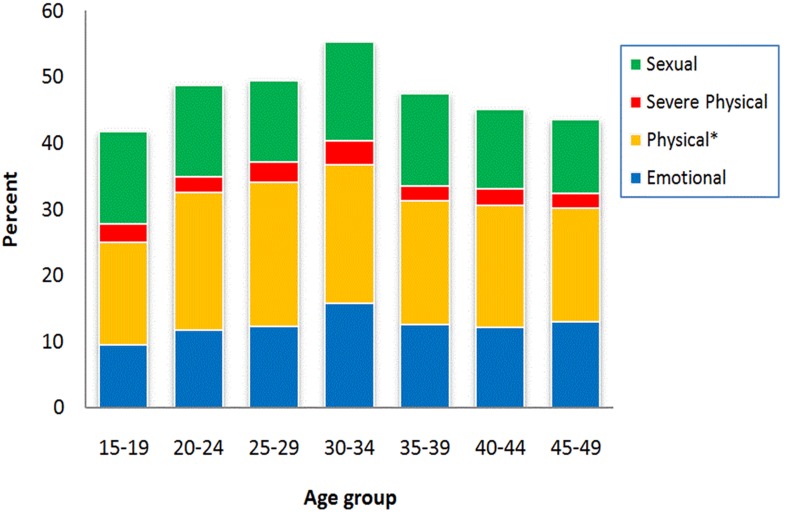


With a few exceptions, patterns of reported IPV were similarly distributed across all age groups and several interesting trends emerged. As depicted in Figure 1 the proportion of emotional IPV increased with age (from 9% to 13%), while the proportion of sexual violence decreased (from 14% to 11%). It is important to note that the percentages of women reporting both emotional and sexual IPV peaked at 30 to 34 years of age with values of 16% and 15%, respectively. The difference in sexual IPV between women in 30-34 age group and the oldest group (45 to 49 years) was the only association found to be statistically significant (p < 0.05).

**IPV and Education**

**Figure 2:**
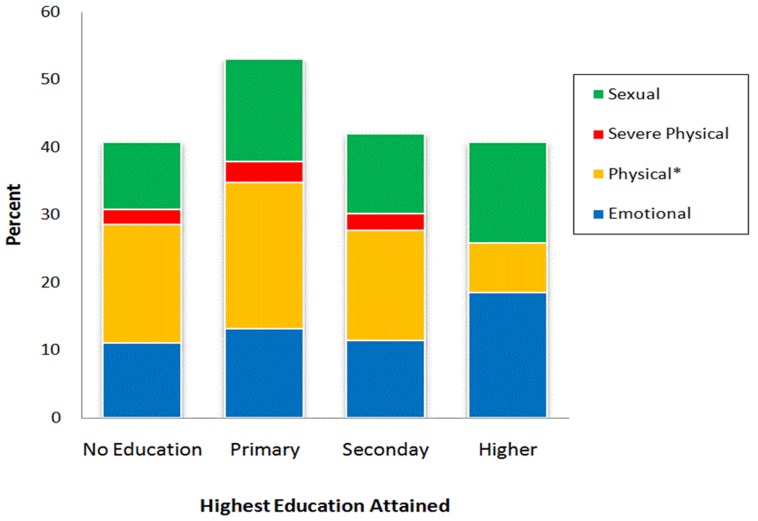


Among women in the no education, primary, and secondary groups, the most frequently reported IPV was ‘being pushed or shaken, slapped or punched’ (16 to 22%), followed by ‘emotional’ IPV (11 to 13%), ‘sexual’ IPV (10 to 15%), and ‘severe physical’ IPV (2 to 3%). Among women with the highest level of education, 7% reported ‘being pushed or shaken, slapped or punched’, 5% reported ‘sexual’ IPV, and 19%, reported ‘emotional’ IPV (Figure 2). These differences were not statistically significant. 

**IPV and Place of Residence**

Eleven percent of women who were living in urban areas and 13% of those who were living in rural areas reported being subject to ‘emotional’ IPV. Being pushed or shaken, slapped or punched was reported by 20% of women both in the urban and rural groups. Also, both groups reported ‘severe’ IPV among 3%, but ‘sexual’ IPV was among 12% and 14%, respectively. No statistically significant association was detected among these groups. 

**Multivariate Associations**

As depicted in Table 2, women ages 15 to 19 were less likely to report ‘emotional’ IPV compared to women ages 45 to 49 (OR = 0.70; 95% CI: 0.49-0.99). Women ages 25 to 29 were more likely to report ‘being pushed or shaken, slapped or punched’ compared to those ages 45 to 49 (OR = 1.35; 95% CI: 1.05-1.73). Furthermore, women ages 30 to 34 were more likely to report ‘sexual’ IPV compared to women ages 45 to 49 (OR = 1.40; 95% CI: 1.03-1.90). Finally, women who could not read at all were less likely to report ‘sexual’ IPV than women who could read a full sentence (OR = 0.76; 95% CI: 0.66-0.87). We did not find any significant difference in the patterns of IPV in women living in rural vs. urban areas.

**Table:** Table 2: **Number of respondents in each category (n), proportion within each category of IPV (% of n) and adjusted ORs and CIs for IPV by socio-demographic characteristics.**

Variable	n	Emotional violence		Pushed or shaken, slapped or punched		Severe violence		Sexual violence	
		% of n	OR: 95% CI	% of n	OR: 95% CI	% of n	OR: 95% CI	% of n	OR: 95% CI
Age									
15-19	753	9.4	0.7:0.49-0.99*	15.6	NS	2.8	NS	13.9	NS
20-24	2257	11.7	NS	20.8	NS	2.4	NS	13.8	NS
25-29	1887	12.2	NS	21.8	1.35: 1.05-1.73*	3.1	NS	12.3	NS
30-34	1295	15.8	NS	20.9	NS	3.6	NS	14.9	1.40: 1.03-1.90*
35-39	874	12.6	NS	18.6	NS	2.3	NS	14.0	NS
40-44	687	12.1	NS	18.5	NS	2.5	NS	11.9	NS
45-49	538	13.0	NS	17.1	NS	2.2	NS	11.2	NS
Total	8291								
Place of residence									
Urban	970	11.1	NS	20.0	NS	2.8	NS	11.6	NS
Rural	7321	12.7	NS	19.9	NS	2.8	NS	13.6	NS
Total	8291								
Literacy Level									
Cannot read at all	3680	11.9	NS	20.3	NS	2.9	NS	11.8	0.76: 0.66-0.87*
Can read partial sentence	747	11.6	NS	19.5	NS	3.1	NS	12.4	
Can read whole sentence	3850	13.1	NS	19.6	NS	2.7	NS	15.0	
Total	8277								
Education Level									
No Education	2316	11.1	NS	17.5	NS	2.2	NS	9.9	NS
Primary	5176	13.2	NS	21.6	NS	3.1	NS	15.1	NS
Secondary	772	11.5	NS	16.2	NS	2.5	NS	11.8	NS
Higher	27	18.5	NS	7.4	NS	0	NS	14.8	NS
Total	8290								

*OR: CI with statistical significance of at least p&0.05

## Discussion

In this study we aimed to examine the lifetime prevalence and predictors of different types of intimate partner violence (IPV) among Malawi women ages 15-49. Based on the demographic data collected, sample characteristics were similar to the demographics of the overall population of women in Malawi.^48,49^

**Prevalence of IPV**

The present study shows that the lifetime prevalence of ‘emotional’ IPV was found to be 13%, which is less than the prevalence reported in other sub-Saharan African countries, such as Uganda (40%),^51^ Zimbabwe (37%),^52^ and Kenya (24%),^16^ (Table 3). Approximately one in every four women interviewed reported being pushed or shaken, slapped or punched, and about 3% reported severe physical violence (i.e., being strangled or burned, threatened with a knife, gun or other weapon). The prevalence of IPV in other countries in the region has often been reported simply as physical IPV, rather than being divided into ‘types of IPV’, and rates are often reported as incidence within the past year. This makes it difficult to compare the findings from this study with other countries; however, we attempt to present some rough comparisons in Table 3. According to this table, the incidence of physical IPV in Botswana, Lesotho, Mozambique, Namibia, Swaziland, Zambia and Zimbabwe ranges between 8 to 27%.27 As for ‘sexual violence’, the rate, in our study, was slightly over 13% which is comparable to the rate in Kenya (14%),^53^ Zimbabwe (37%)^52^and pregnant women in Uganda (37%).^54^

**Table:** Table 3: **IPV in Malawi and the Neighboring Countries **

Country	Definition of IPV	Life time Prevalence	12-months
Malawi	Emotional IPV	13%	
	Pushed, shaken, slapped or punched	20%	
	Severe physical abuse	3.0%	
	Sexual	13%	
Uganda^51-54^	Verbal and physical threats	-	40%
	Physical abuse	30%	
	Sexual IPV	37%	
Zimbabwe^4^	Psychological	37%	17%
	Physical	32%	
	Sexual IPV		
Kenya^14^	Emotional,	-	24%
	Physical		38%
	Sexual abuse		14%
Botswana^74^	Beat, kicked or slapped	-	21%
	Forced sex		10.3%
Lesotho^15^	Beat, kicked, or slapped	-	12%
Zambia^15^	Beat, kicked, or slapped	-	27%
Mozambique^15^	Beat, kicked, or slapped	-	8%
Nambia^15^	Beat, kicked, or slapped	-	15%
Malawi^15^	Beat, kicked, or slapped	-	6%

While our data suggests that IPV is common in Malawi, it appears to be less prevalent than other southern African countries. More studies are needed to investigate and clarify if men in Malawi indeed are less likely to commit acts of physical IPV and if so, why or what are the determining factors? For decades, patriarchal ideology in Malawi has allowed men to exercise power over women, which makes IPV normative in this culture (i.e. part of men’s role to ‘correct’, and ‘discipline’ women).^55,56^ Nonetheless, lower rates of IPV in Malawi could be the result of an addendum to the constitution of the Malawian Republic in 2004 that provided full protection of women’s right against discrimination on the basis of their gender^57^ and ever since, gender-related empowerment policies have slowly been enforced and criminal penalties for abuse has gain greater media attention.^58^

The lower rate of physical IPV in Malawi could also be the result of under-reporting by women due to their fear of stigma and retribution, lack of confidentiality in treatment facilities, and Malawi’s practice of traditional laws and weak policies^59,60^ that promote the belief that IPV should be dealt privately and within the family,^61^ and therefore, reporting IPV abuse is the sign of a woman’s disloyalty to her husband.^62^ One finding from Malawi indicates that only 4% of the women who have experienced IPV reported it to the police, and those who do report are not aware of their legal rights.^58^

Our results also indicate that the percentage of women who reported severe physical violence was less than those who reported sexual violence. Yet, findings from previous studies suggest that sexual violence is a marker for severity of violence, which means that women who have experienced sexual IPV have also experienced severe physical and emotional violence.^63^ Similar results have been reported from a study in Indonesia.^64^ To explain this variation, Garcia-Moreno and colleagues suggest that cultural differences could be the determining factor in defining whether it is acceptable for men to control or chastise their women.^46,65^ Based on the principle of ecological theory we can link perception of sexual violence in a relationship to the broader social environment, including cultural views of sexual IPV and family settings or circumstances. This could explain why a woman may view her experience of abuse as nothing inherently wrong and therefore not report it. In a study from Malawi, only 27%, 19%, and 17% of women who experience sexual, economic, and physical abuse, thought it was legally wrong.^58^

Therefore, exploring and comparing the attitudes and behaviors of Malawian women towards different types of IPV and their levels of acceptability of IPV across different socio-demographic or cultural groups may shed some light in this direction and help interventionists in reducing rates of IPV in Africa and other regions across the globe.

In this study the interviews were conducted in the native language and questionnaires were translated and back-translated to avoid language error and barriers. However, one can still speculate that, for example, the lower reported rates of emotional IPV in the study could be the result of women’s misunderstanding or inability to comprehend questions related to emotional IPV. More methodological studies in this respect are needed. 

**Age and IPV**

Among the socio-demographics included in this study, age emerged as the most interesting variable. Three statistically significant findings were related to age. First, women in the youngest age group were less likely than those in the oldest age group to report ‘emotional’ IPV. This may represent a cumulative effect with more women in the oldest age group experiencing more ‘emotional’ IPV. It could also be that ‘emotional’ IPV increases with age due to increased conflict in the household, possibly related to children, financial responsibilities or marital discord. Or it could reflect a cultural shift in Malawi towards younger women gaining more power as a result of pursuing education, employment, and economic independence – all contributing to less vulnerability to and acceptance of emotional abuse.^26^ Second, women ages 25 to 29 were more likely than women in the oldest group to report being pushed or shaken, slapped or punched; possibly indicating an increase in physical violence among younger women (p < 0.05). This differs slightly from a study of other southern African countries (i.e., Botswana, Lesotho, Mozambique, Namibia, Swaziland, Zambia and Zimbabwe) where researchers found no significant difference in physical IPV with respect to age, but found trends showing higher rates among women ages 30 to 39 and somewhat lower rates among the younger and older groups.^27^ Third, women ages 30 to 34 were significantly more likely to report sexual IPV than women in the oldest age group. Again, this may reflect an increase in sexual IPV among younger women, or, perhaps, a reluctance to report sexual IPV among older women. Few studies have addressed potential associations between age and IPV and those have generally found no statistically significant differences.^27,51,54^ Methodological differences also complicate the interpretation of such studies as age groups are defined in several ways, outcomes may relate to general or specific types of IPV, trends may be expected to be linear with increasing age (rather than variable, as this study found), and study populations differ vastly by geographic and cultural factors. 

**Literacy and IPV**

In our study, women who were completely illiterate were significantly less likely to report sexual IPV than their peers who could read a full sentence. Examining the trends that emerged among women in various education levels, it appears that sexual IPV may be more prevalent among women with a higher level of education. Due to the small number of women in the higher education group, these associations were not statistically significant.

Other studies have shown a lower overall prevalence of IPV among sub-Saharan African women with higher levels of education compared to those with little or no education.^66^ However, more research is needed to explore the relationship between sexual IPV and education level in Malawi women. These studies can test the assumptions of the bargaining model that is rooted in exchange theory. According to this model, the experience of IPV among women decreases as they gain greater control over economic resources.^67^ It is likely that women with a higher level of education will have higher access to resources and therefore less tolerant of an abusive relationship. On the other hand, proponents of resource theory, consistent with the bargaining framework, argue that women’s economic gains can spark a “backlash” depending on the husband’s gender ideology; a situation in which men’s frustrations with lack of economic resources becomes a risk for abuse of the women.^68,69^ Therefore, it is also possible that higher education makes these women more threatening to their male partners, and sexual IPV may reflect an attempt by the male partner to gain more power or control over the woman. To the same extent, more methodological work is needed to explore whether lower levels of IPV among the less literate women in this study has something to do with their economic insecurity and dependency, or psychological attachment to their husband/partner, or social/cultural norms, or all of the above. As a result of any of these alternative explanations rural women may develop different perceptions or reactions to what is considered an ‘emotionally abusive’ relationship by the larger group. This could be the basis for future research.

This study did not look at the differences in the education levels between partners, which could be another factor in the association between education and sexual IPV. This is one more potential area for future investigation.

**Location of Residence and IPV**

Surprisingly, we could not find any significant difference in reported IPV of any type between women residing in rural versus urban areas. Previous studies have shown a higher prevalence as well as a more accepting attitude toward various types of IPV in rural areas compared to urban areas, possibly because traditional gender norms are more prevalent in rural areas.^1,21^ Many studies, however, fail to compare IPV in urban and rural areas, though they often contain data from both settings. Given that only 12% of the respondents in this study were from urban areas in Malawi, it is possible that more data from women living in urban areas is necessary for differences to emerge. It is also possible that in rural areas there are other factors such as local or tribal customs and religious backgrounds that may complicate the patterns observed. Examining differences in reported prevalence between different rural areas might help to elucidate such confounders. 

Different approaches and interventions may be needed to address IPV in rural areas where anti-discrimination laws are less enforced than in the urban areas.^70^ These interventions can benefit from identifying local members and leaders and eliciting their opinions in how to target traditional customs such as patriarchal heritage, wife inheritance, and polygamy, customs which are more practiced and accepted in the rural areas.^70^ Such a community-participatory approach can facilitate incorporating women’s voices and perspectives into women-centered programs to reduce gender-based IPV. It also can link IPV-prevention programs to the formal and informal local institutions and NGOs in supporting and improving women local access to agricultural training, basic education and health facilities as well as social and legal IPV-related services. Such collaboration can further support the sustainability of these programs.^71^

**Limitations**

Although the study included a large number of respondents, relatively small samples in some socio-demographic groups may have prevented any possible observed associations from reaching statistical significance. With small samples, the possibility that the emerging trends are inaccurate or not representative of the larger population cannot be excluded. Prevalence data was only available to the authors according to specific types, periods, and frequencies of IPV, preventing an overall estimate of the general prevalence of IPV (of any type) in Malawi. 

The original study design sought to minimize selection bias by systematically selecting a large number of households in multiple districts in both urban and rural areas. The questionnaire was administered by trained personnel in accordance with WHO recommendations and participant confidentiality was ensured, minimizing measurement bias. Nevertheless, reporting bias is possible, as women may not have accurately reported their experiences with IPV.^16^

In the surveys the interviewers asked participating women a series of questions related to the demographic, family, individual and personal attitudes, such as background characteristics (age, education, religion, assets, etc.); reproductive history; knowledge and use of family planning methods; antenatal, delivery, and postnatal care; infant and child health including feeding practices; sexual and marital activities; and household autonomy and domestic violence. The trained, experienced interviewers gained the confidence of the female respondents and secured maximum disclosure of information. After answering all the previous questions, generally women respondents felt confident to answer violence related questions. That is one of the vital reasons for a high response rate to domestic violence questionnaires in all demographic and health surveys including MDHS.

Despite the efforts of the MDHS interviewers to maintain confidentiality and encourage accurate reporting, IPV remains a very sensitive topic. Women may have felt scared or ashamed to reveal such personal and emotionally damaging information, leading to possible underreporting of IPV. However, as mentioned above the IPV questionnaires were asked within the context of several other questions to enhance trustworthiness and maximum disclosure of IPV facts.^39^ Additionally, the data collected were specific to married and cohabitating women, while the true prevalence of IPV may be higher once we include lifetime prevalence of IPV among women who were divorced or left their partner to avoid IPV. In addition, this study did not address IPV perpetrated by women against men, or IPV within various types of same-sex relationships, further underestimating the true prevalence of IPV in Malawi. 

**Future Directions **

The findings presented in this study help to provide a background and framework for important future research and interventions to address IPV in Malawi. The next step is to attempt to understand the social and cultural norms surrounding acceptability and justification of IPV,^72^ as well as willingness to disclose IPV among women in Malawi, and how they are different in other countries in sub-Saharan African countries. Demonstration of cultural acceptance of IPV would highlight the importance of interventions aimed at changing attitudes and societal norms. Moreover, policy changes and increased recognition and service provision by health care facilities are legitimate and valuable strategies to combat IPV. For better policy formulation for violence prevention, more studies on IPV, including cultural perspective in Malawi, are warranted. 

Indeed, if there is a sincere desire to prevent the onset of IPV (i.e., primary prevention) in Malawi or any other parts of the world, IPV-related laws should allow for prosecuting IPV perpetrators, male or female. Additionally educating local people about the cycle of violence, for example through media campaigns, can increase their awareness in reference to the negative consequences of IPV on health, family, and community. It further can enhance public knowledge in how to communicate and seek help.^73^ To reduce existing IPV (i.e. secondary prevention), health care providers should not only be trained to screen, identify and treat IPV-related cases, they should also be cognizant of their legal responsibility to document, collect forensic evidence, and report such cases to authorities.^73^ In addition, efforts are needed to align existing IPV-related laws with law enforcement to facilitate the prosecution of IPV perpetrators. 

## Conclusion

Worldwide, women are victims of IPV and all of its associated detrimental consequences. This study shows that at least one in five women in Malawi experience some level of physical IPV and that over one in ten women report experiencing emotional and sexual IPV. Although this represents a slightly lower prevalence than other sub-Saharan African countries, discrepancies in the assessment tool, the definition of IPV, the population under study and the design of the study warrants caution in comparing our findings to others’. Nevertheless our findings affirm that IPV is indeed a pressing public health and human rights issue in Malawi and highlights the need for further research and interventions. In this study, younger age appeared to be a protective factor against emotional IPV but a risk factor against physical and sexual IPV, while older women were more at risk for emotional IPV. Education and literacy levels also appeared to protect women from exposure to physical IPV but not so much against sexual IPV. We found, however, no difference in the prevalence of IPV according to place of residence (rural versus urban). More research is needed to further explore these patterns and to examine cultural attitudes including acceptability and justifying attitudes towards IPV in Malawi, ultimately providing the background needed for effective, targeted interventions. 
